# A cross-sectional survey assessing factors associated with reading cancer screening information: previous screening behaviour, demographics and decision-making style

**DOI:** 10.1186/s12889-017-4224-9

**Published:** 2017-04-18

**Authors:** Alex Ghanouni, Cristina Renzi, Jo Waller

**Affiliations:** Health Behaviour Research Centre, Department of Epidemiology and Public Health, University College London, Gower Street, London, WC1E 6BT UK

**Keywords:** Cancer screening, Population screening, Public education, Decision making, Informed choice, Survey, England

## Abstract

**Background:**

There is broad agreement that cancer screening invitees should know the risks and benefits of testing before deciding whether to participate. In organised screening programmes, a primary method of relaying this information is via leaflets provided at the time of invitation. Little is known about why individuals do not engage with this information. This study assessed factors associated with reading information leaflets provided by the three cancer screening programmes in England.

**Methods:**

A cross-sectional survey asked screening-eligible members of the general population in England about the following predictor variables: uptake of previous screening invitations, demographic characteristics, and ‘decision-making styles’ (i.e. the extent to which participants tended to make decisions in a way that was avoidant, rational, intuitive, spontaneous, or dependent). The primary outcome measures were the amount of the leaflet that participants reported having read at their most recent invitation, for any of the three programmes for which they were eligible. Associations between these outcomes and predictor variables were assessed using binary or ordinal logistic regression.

**Results:**

After exclusions, data from 275, 309, and 556 participants were analysed in relation to the breast, cervical, and bowel screening programmes, respectively. Notable relationships included associations between regularity of screening uptake and reading (more of) the information leaflets for all programmes (e.g. odds ratio: 0.16 for participants who never/very rarely attended breast screening vs. those who always attended previously; *p* = .009). Higher rational decision-making scores were associated with reading more of the cervical and bowel screening leaflets (OR: 1.13, *p* < .0005 and OR: 1.11, *p* = .045, respectively). Information engagement was also higher for White British participants compared with other ethnic groups for breast (OR: 3.28, *p* = .008) and bowel (OR: 2.58, *p* = .015) information; an opposite relationship was observed for older participants (OR: 0.96, *p* = .048; OR: 0.92, *p* = .029).

**Conclusions:**

Interventions that increase screening uptake may also increase subsequent engagement with information. Future research could investigate how to improve engagement at initial invitations. There may also be scope to reduce barriers to accessing non-English information and alternative communication strategies may benefit participants who are less inclined to weigh up advantages and disadvantages as part of their decision-making.

## Background

Public health policymakers, academics, and practitioners broadly accept the principle that cancer screening invitees should be able to consider the risks and benefits of being tested and make informed decisions about their participation [1–3]. In the UK, the primary method by which organised National Health Service (NHS) cancer screening programmes aim to achieve this is via information leaflets, generally posted to eligible individuals alongside an invitation to have a test (typically mammography for breast screening, cytology or human papillomavirus testing for cervical screening, or stool testing for bowel screening [4–6]).

The national scale of cancer screening programmes means that mailed leaflets can be the only practical method of communicating with all invitees, which amounts to millions of people every year. Consequently, an important outcome related to informed choice is the extent to which screening invitees read this information as it is often a prerequisite for achieving a satisfactory level of understanding. In addition, invitees who do not read screening information and are not screened are unlikely to have made an informed decision not to participate. These individuals may miss out on the health benefits of screening as a result of their lack of engagement.

Previous research on screening information has often focused on its content and quality (e.g. [7]), whereas the extent to which people engage with this information is relatively understudied. Recent work suggests that lack of engagement is a significant barrier to informed choice: Kobayashi et al. [8] report that approximately 20% of individuals eligible for bowel cancer screening in England have never read any of the associated leaflet and this figure is 63% among those eligible who had never been screened (vs. 4% of individuals who had been screened at least once). Non-white individuals are also less likely to have read any of the information leaflet. However, there may be other important factors associated with information engagement that have not so far been investigated. For example, unmarried individuals have lower screening uptake than married individuals [9–11], as do those who do not already know someone diagnosed with cancer [12–17]; this may imply a lesser degree of engagement with information materials in these groups.

Psychological factors may also be associated with (not) reading the leaflet, such as how people tend to make important decisions. One of the most commonly used methods of categorising “decision-making styles” (DMS) is based on five dimensions (avoidant, dependent, intuitive, rational, and spontaneous [18]) and it is plausible that more rational individuals (those who tend to weigh up the advantages and disadvantages before making a decision) are more likely to read information that is designed to be concordant with this way of thinking, whereas the opposite may be true for more avoidant individuals (those who tend to procrastinate before making decisions). DMS may suggest opportunities for improving informed choice in the context of cancer screening although, to our knowledge, they have not previously been investigated. For example, if more dependent individuals (those who rely on others when making decisions) are less likely to read information, they may benefit from greater involvement of people they trust (e.g. healthcare professionals) in order to provide screening information effectively.

We used a large survey of the general population to explore the extent to which screening behaviour, demographic characteristics, and DMS were associated with reading information leaflets for the breast, cervical, and bowel cancer screening programmes in England.

## Method

### Design

Data were collected as part of the Attitudes, Behaviour and Cancer UK Survey (ABACUS; wave 3) in April and May, 2016. A market research agency (Taylor Nelson Sofres; TNS) collected data during a weekly omnibus survey via face-to-face computer-assisted interviews.

### Participants

Following institutional ethical approval, recruitment was carried out in England using random location sampling based on Census statistics and the Postcode Address File. Quotas were set for key demographic characteristics (e.g. gender and employment status) to obtain a nationally representative sample. Interviews were carried out with a total of 2111 individuals aged 18–70 years in their homes, following their verbal consent to participate (in accordance with standard survey methodology, responding to questions constituted consent).

### Measures

#### Demographics

Basic demographic data were collected regarding participants’ gender, marital status, ethnicity, highest level of education qualification obtained, social class grade, and age. Participants were also asked to indicate i) whether they knew anyone who had been diagnosed with cancer and ii) whether they had been diagnosed with cancer themselves; those that had were asked to state which organ it affected (bowel, lung, breast, cervix, prostate, or other).

#### Decision-making styles

Measures of participants’ tendencies towards using particular decision-making styles were preceded with the following information:

“*We would like to start by asking you some questions about how you make important decisions. I am going to read out some statements describing how individuals go about making important decisions and I would like you to tell me how much you agree or disagree with each one.”*

This was followed by 25 items designed by Scott and Bruce [18], relating to five subscales of decision-making styles and consisting of five items each: ‘rational’ (e.g. *“My decision-making requires careful thought”*), ‘intuitive’ (e.g. *“I generally make decisions that feel right to me”*), ‘spontaneous’ (e.g. *“I make quick decisions”*), ‘avoidant’ (e.g. *“I postpone decision making whenever possible”*), and ‘dependent’ (e.g. *“I rarely make important decisions without consulting other people”*). Items were presented in a random order for each participant. Responses were recorded on a five-point Likert scale of “*strongly disagree”* to *“strongly agree”*.

The measure has previously been reported to possess content, concurrent, and construct validity [18]. Acceptable reliability was demonstrated; Cronbach’s α was calculated for the five subscales across all participants recruited for this wave of ABACUS and values ranged from 0.72 to 0.80. Internal consistency could not be substantially improved by omitting items from any subscales.

#### Screening participation

Individuals were presented with up to three items relating to their previous screening participation if they met the eligibility criteria for one of the three cancer screening programmes offered by the NHS in England (breast screening: women aged 50–70 years; cervical screening: women aged 25–64 years; bowel screening: men and women aged 60–70 years). These items were omitted if participants were anticipated to find the question upsetting or irrelevant (e.g. they were only asked about previous participation in breast cancer screening if they had not been diagnosed with breast cancer). Items were preceded by text similar to the following:

*“The next set of questions are about breast screening. The NHS breast screening programme invites women to have regular mammograms (x-rays of their breasts).”*

The first item was based on the Precaution Adoption Process Model [19], a stage model of protective health behaviour. The model categorises people as being in one of seven stages from *“unaware”* to *“maintenance”*. Participants were asked, *“Which one of the following best describes you?”*, followed by a choice of one of the following response options:

*“I have never heard of breast screening/I have heard of breast screening but have never been invited/I have been invited to breast screening but have never been/I have been invited to breast screening but have not been every time I was invited/I have been invited to breast screening and have been every time I was invited”*

Participants who stated they had previously been invited but had not been every time were asked whether they had been screened the last time they were invited (*“Yes”*/*“No”*/*“Don’t know”*) and also asked, *“Before the last time you were invited, how regularly had you been going for breast screening?”*. Response options consisted of:

*“Every time I was invited (including if you have only been invited once before your last invitation)/Most times I was invited/Some of the times I was invited/Very rarely or never/I have never been invited before my most recent invitation”* (the first option was excluded for participants who had been screened at their last invitation, in order to prevent incompatible responses).

**Fig. 1:**
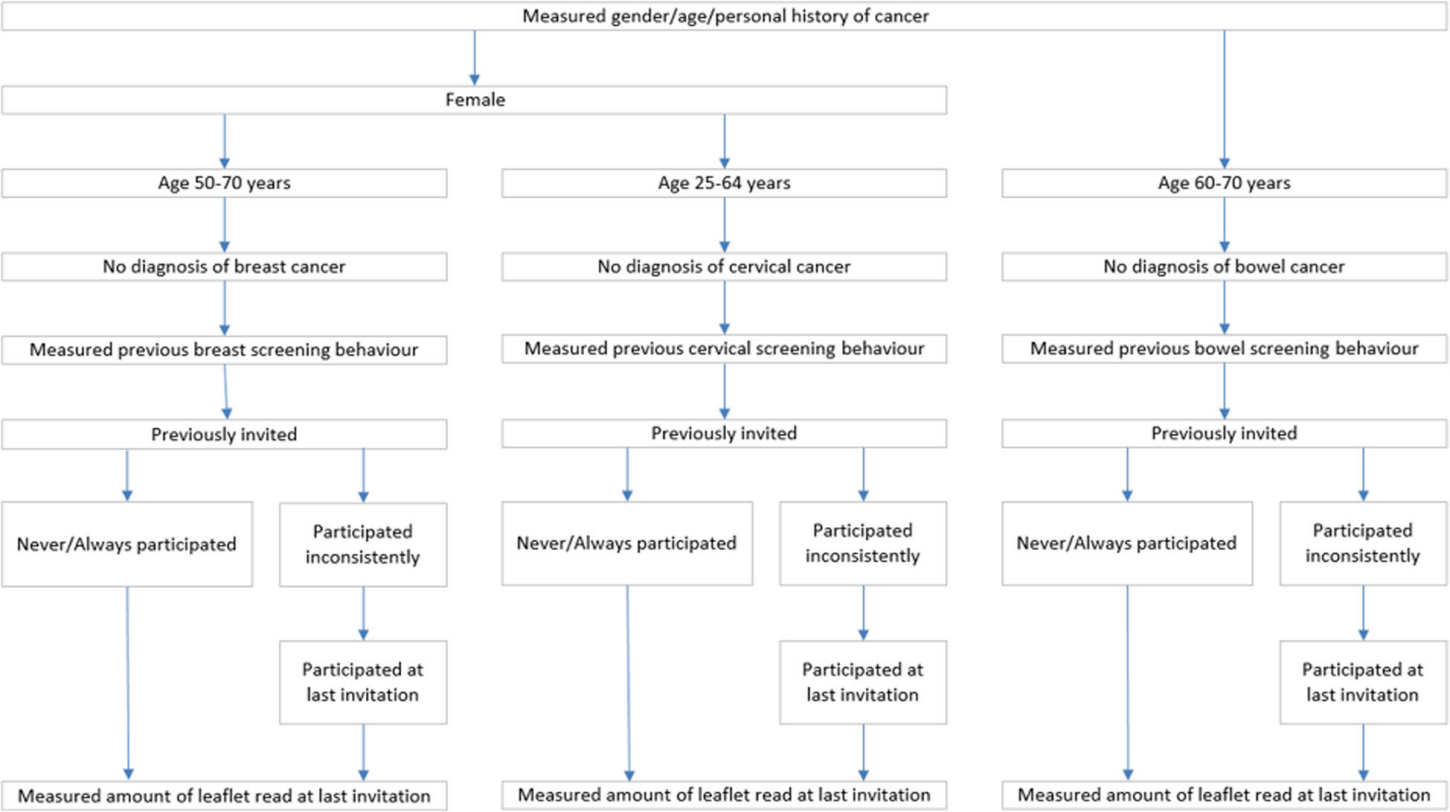
Flow of participants through questions relating to screening behaviour and reading the leaflet(s)

The flow of participants through this section is summarised in Fig. 1.

#### Reading information leaflets at participants’ most recent screening invitation

Participants who reported having previously been invited to screening were presented with one item based on a previous survey [8]: *“When people are invited for breast screening they are sent an NHS leaflet about the screening programme and test. Thinking about the last time when you were invited for cervical screening, how much of the information leaflet did you read?”* Response options consisted of: *“None of it/Some of it/About half of it/Most of it/All of it/I don’t remember seeing a leaflet”*.

### Piloting

The survey was piloted using a series of cognitive interviews [20], carried out by the research team with lay members of the public (*n* = 11), to ensure that items were understood as intended and that they did not cause undue participant burden or distress. This was followed by a ‘soft-launch’ of the survey to a small number of the eligible sample (*n* = 431) by the market research company to determine whether scales had sufficient reliability and that programming was successfully routing participants to all appropriate questions.

### Analysis

Data cleaning and recoding was carried out using SPSS 22 for Windows (IBM, Armonk, NY, USA). Exclusion criteria were ineligibility due to i) gender, ii) age, iii) previous diagnosis of relevant cancer, iv) reporting a non-ordinal highest level of education (e.g. *“BTEC/ONC”*), and v) reporting not having previously been invited to screening or not having seen an information leaflet. Participants with missing data were also excluded i.e. pertaining to cancer history (theirs or others they know), decision-making items, screening history, or demographic characteristics. Exclusions due to missing data were limited (7.4% to 10.4% of otherwise eligible cases) and hence imputation was not attempted.

Responses to decision-making items were scored from one to five (higher scores representing stronger agreement) and summed for each of the five subscales. Responses to the three items on previous screening history were recoded to form a measure of regularity of screening participation: participants were coded as having very rarely or never participated in screening before their most recent invitation, participated some or most of the time, or always. Highest level of education was categorised based on an approach used by the Office of National Statistics [21]: Qualifications below degree level i.e. *“O Levels or GCSE equivalent (Grades D-G)”*, *“O Levels or GCSE equivalent (Grades A-C)”*, *“A Levels or Highers”*, *“Higher education below degree”* were coded as *“Approximately Level 1, 2, or 3”*, and a university degree or higher i.e. *“Bachelor’s degree”* or *“Further degree or higher (*e.g. *Master’s, PhD,* etc.*)”* were coded as *“Approximately Level 4”*. The remaining category was *“No formal qualifications”*. Social class grade was categorised as *“Grades A or B”*, *“Grades C1 or C2”*, and *“Grades D or E”*. Due to the small numbers in each sub-category, ethnicity and marital status were dichotomised (*“White British”* vs. *“Other ethnic groups”* and *“Married or living as a couple”* vs. *“Single, widowed, divorced, or separated”*).

Separate sets of results were generated for the three cancer screening programmes. Descriptive statistics were used to characterise participants eligible for each one. The main hypotheses tested were whether screening behaviour, demographic characteristics, and DMS scores were associated with our outcome of interest, i.e. the amount of the information leaflet that participants had read at their last invitation. The assumption of proportional odds was violated for the analysis of reading the breast leaflet (test of parallel lines: *p* < .0005) and a binary logistic regression model was generated for this screening programme, in which the outcome was dichotomised into reading all of the leaflet vs. reading less than all of the leaflet. Sensitivity analyses examined three other possible thresholds for dichotomisation i.e. i) reading all/most of the leaflet vs. reading less; ii) reading all/most/about half the leaflet vs. reading less, and iii) reading at least some of the leaflet vs. reading none of it. The assumption of proportional odds was not rejected for the analysis of reading the cervical (*p* = .128) or bowel screening leaflets (*p* = .376) and hence ordinal logistic regression was carried out for these two programmes.

Predictor variables consisted of gender (where applicable), marital status, ethnicity, education, social class grade, personal diagnosis of cancer, knowing someone close with a previous diagnosis of cancer, age, regularity of screening participation (for the relevant programme), and scores on the five DMS subscales. There was little evidence of (multi-)collinearity between independent variables (all Variance Inflation Factors <2.34) and a Box-Tidwell procedure for the model of reading the breast screening leaflet showed no violations of the assumption of linearity (all *p*-values > .240).

Crude proportions (e.g. of White British vs. Other ethnic groups reporting reading each possible amount of the leaflet) and means (and standard deviations; SDs) are reported along with adjusted odds ratios (ORs) for reading all of the leaflet vs. reading less than all of it, or being in a “higher” category of reading the leaflet (e.g. reading some of the leaflet vs. reading less than some or reading most of the leaflet vs. reading less than most), taking into account all predictor variables.

## Results

### Participant characteristics

Table 1, Table 2, and Table 3 describe the main characteristics of participants included in each analysis. Appendix 1 shows the numbers and percentages of participants excluded from the analyses, along with each reason.

**Table 1:** Reading breast screening information (women only): descriptive statistics, adjusted ORs, 95% CIs, *p*-values for variables in the multivariable binary logistic regression model

	Amount read of the breast screening leaflet: n (%)/M (SD)						Adjusted OR, 95% CI	*p*-value
	None of it	Some of it	About half of it	Most of it	All of it	Total	Read all of the leaflet	
Characteristic	(*n* = 28; 10.2%)	(*n* = 43; 15.6%)	(*n* = 13; 4.7%)	(*n* = 44; 16.0%)	(*n* = 147; 53.5%)	(*n* = 275)	(vs. read less than all)	
Ethnicity								
White British	25 (10.2)	37 (15.0)	10 (4.1)	37 (15.0)	137 (55.7)	246	3.28, 1.36 to 7.89	**.008**
vs. Other ethnic groups	3 (10.3)	6 (20.7)	3 (10.3)	7 (24.1)	10 (34.5)	29		
Marital status								
Married or living as a couple	15 (8.4)	30 (16.9)	9 (5.1)	29 (16.3)	95 (53.4)	178	0.80, 0.45 to 1.42	.802
vs. Single, widowed, divorced, or separated	13 (13.4)	13 (13.4)	4 (4.1)	15 (15.5)	52 (53.6)	97		
Highest level of education							Overall: .150	
No formal qualifications	11 (12.5)	15 (17.0)	6 (6.8)	20 (22.7)	36 (40.9)	88	0.44, 0.19 to 1.01	.052
Approximately Level 1, 2, or 3	13 (10.5)	15 (12.1)	4 (3.2)	21 (16.9)	71 (57.3)	124	0.60, 0.29 to 1.24	.168
vs. Approximately Level 4	4 (6.3)	13 (20.6)	3 (4.8)	3 (4.8)	40 (63.5)	63		
Social class grade							Overall: .876	
Grade A or B	7 (11.3)	8 (12.9)	4 (6.5)	8 (12.9)	35 (56.5)	62	0.90, 0.40 to 2.07	.811
Grade C1 or C2	9 (7.4)	20 (16.5)	6 (5.0)	16 (13.2)	70 (57.9)	121	1.08, 0.57 to 2.05	.811
vs. Grade D or E	12 (13.0)	15 (16.3)	3 (3.3)	20 (21.7)	42 (45.7)	92		
Personal diagnosis of cancer								
Yes	3 (14.3)	1 (4.8)	1 (4.8)	4 (19.0)	12 (57.1)	21	0.90, 0.35 to 2.35	.834
vs. No	25 (9.8)	42 (16.5)	12 (4.7)	40 (15.7)	135 (53.1)	254		
Knows someone with cancer								
Yes	21 (10.4)	32 (15.8)	7 (3.5)	29 (14.4)	113 (55.9)	202	1.23, 0.69 to 2.20	.493
vs. No	7 (9.6)	11 (15.1)	6 (8.2)	15 (20.5)	34 (46.6)	73		
Breast screening regularity							Overall: **.014**	
Never or very rarely attended…	6 (35.3)	4 (23.5)	2 (11.8)	2 (11.8)	3 (17.6)	17	0.16, 0.04 to 0.63	**.009**
Attended some or most of the time…	1 (6.3)	4 (25.0)	2 (12.5)	3 (18.8)	6 (37.5)	16	0.43, 0.14 to 1.30	.135
vs. Always attended before most recent invitation	21 (8.7)	35 (14.5)	9 (3.7)	39 (16.1)	138 (57.0)	242		
Age (in years)	60.1 (5.7)	61.7 (6.4)	59.7 (5.5)	62.2 (5.4)	59.8 (6.1)	60.5 (6.0)	0.96, 0.92 to 1.00	**.048**
Decision-making styles								
Avoidant score	12.3 (3.6)	14.0 (4.2)	14.9 (5.2)	12.6 (3.2)	12.5 (3.6)	12.8 (3.8)	0.97, 0.89 to 1.05	.452
Dependent score	16.0 (3.9)	17.3 (3.5)	17.6 (4.6)	16.1 (4.4)	16.5 (3.8)	16.6 (3.9)	1.03, 0.96 to 1.11	.434
Intuitive score	19.1 (2.5)	18.2 (2.4)	18.6 (3.3)	19.3 (2.7)	18.8 (2.8)	18.8 (2.7)	1.01, 0.91 to 1.12	.892
Rational score	19.1 (3.0)	20.0 (2.0)	19.5 (3.0)	19.1 (2.9)	19.9 (2.9)	19.7 (2.8)	1.08, 0.97 to 1.19	.151
Spontaneous score	14.9 (4.0)	13.4 (3.3)	15.2 (4.7)	15.2 (4.7)	13.8 (3.9)	13.9 (3.7)	1.03, 0.95 to 1.11	.528

Adjusted ORs and 95% CIs are per unit increase in the case of continuous variables; *p*-values <.05 are in bold; all predictor variables are included in the model

**Table 2:** Reading cervical screening information (women only): descriptive statistics, adjusted ORs, 95% CIs, *p*-values for variables in the multivariable ordinal logistic regression model

	Amount read of the cervical screening leaflet: n (%)/M (SD)						Adjusted OR, 95% CI	*p*-value
	None of it	Some of it	About half of it	Most of it	All of it	Total	Read more of the leaflet	
Characteristic	(*n* = 94; 16.9%)	(*n* = 108; 19.4%)	(*n* = 34; 6.1%)	(*n* = 106; 19.1%)	(*n* = 214; 38.5%)	(*n* = 556)	(i.e. any amount more)	
Ethnicity								
White British	85 (19.5)	84 (19.3)	19 (4.4)	78 (17.9)	169 (38.9)	435	0.79, 0.53 to 1.18	.248
vs. Other ethnic groups	9 (7.4)	24 (19.8)	15 (12.4)	28 (23.1)	45 (37.2)	121		
Marital status								
Married or living as a couple	61 (16.1)	73 (19.3)	25 (6.6)	70 (18.5)	150 (39.6)	379	1.11, 0.79 to 1.56	.558
vs. Single, widowed, divorced, or separated	33 (18.6)	35 (19.8)	9 (5.1)	36 (20.3)	64 (36.2)	177		
Highest level of education							Overall: .850	
No formal qualifications	14 (19.4)	12 (16.7)	4 (5.6)	15 (20.8)	27 (37.5)	72	0.92, 0.52 to 1.64	.784
Approximately Level 1, 2, or 3	51 (17.2)	57 (19.3)	19 (6.4)	55 (18.6)	114 (38.5)	296	1.06, 0.73 to 1.53	.770
vs. Approximately Level 4	29 (15.4)	39 (20.7)	11 (5.9)	36 (19.1)	73 (38.8)	188		
Social class grade							Overall: .142	
Grade A or B	20 (15.4)	29 (22.3)	6 (4.6)	22 (16.9)	53 (40.8)	130	0.77, 0.47 to 1.26	.297
Grade C1 or C2	52 (18.1)	59 (20.6)	17 (5.9)	57 (19.9)	102 (35.5)	287	0.67, 0.44 to 1.00	.050
vs. Grade D or E	22 (15.8)	20 (14.4)	11 (7.9)	27 (19.4)	59 (42.4)	139		
Personal diagnosis of cancer								
Yes	4 (14.8)	4 (14.8)	1 (3.7)	4 (14.8)	14 (51.9)	27	1.27, 0.60 to 2.68	.537
vs. No	90 (17.0)	104 (19.7)	33 (6.2)	102 (19.3)	200 (37.8)	529		
Knows someone with cancer								
Yes	67 (18.0)	76 (20.4)	20 (5.4)	63 (16.9)	147 (39.4)	373	0.87, 0.62 to 1.22	.420
vs. No	27 (14.8)	32 (17.5)	14 (7.7)	43 (23.5)	67 (36.6)	183		
Cervical screening regularity							Overall: **.023**	
Never or very rarely attended…	14 (27.5)	11 (21.6)	5 (9.8)	6 (11.8)	15 (29.4)	51	0.51, 0.30 to 0.87	.**014**
Attended some or most of the time…	9 (15.5)	12 (20.7)	12 (20.7)	8 (13.8)	17 (29.3)	58	0.68, 0.41 to 1.12	.131
vs. Always attended before most recent invitation	71 (15.9)	85 (19.0)	17 (3.8)	92 (20.6)	182 (40.7)	447		
Age (in years)	43.0 (10.8)	42.7 (11.1)	43.0 (11.8)	42.8 (11.2)	44.7 (11.3)	43.5 (11.2)	1.01, 1.00 to 1.03	.144
Decision-making styles								
Avoidant score	13.6 (4.1)	14.0 (3.9)	14.6 (4.1)	13.9 (3.4)	13.0 (3.8)	13.6 (3.8)	0.97, 0.93 to 1.02	.211
Dependent score	16.8 (4.1)	17.4 (3.1)	18.0 (3.1)	17.0 (3.5)	17.0 (3.4)	17.1 (3.5)	1.00, 0.95 to 1.05	.984
Intuitive score	18.9 (2.5)	18.8 (2.3)	18.5 (2.6)	19.0 (2.7)	18.7 (2.9)	18.7 (2.7)	0.99, 0.93 to 1.05	.663
Rational score	18.7 (3.2)	19.4 (2.0)	19.9 (2.3)	19.8 (2.3)	20.0 (2.8)	19.6 (2.6)	1.13, 1.06 to 1.21	**<.0005**
Spontaneous score	14.8 (3.7)	14.2 (3.5)	15.4 (3.8)	14.7 (3.3)	14.2 (3.6)	14.5 (3.6)	1.02, 0.97 to 1.07	.466

Adjusted ORs and 95% CIs are per unit increase in the case of continuous variables; *p*-values <.05 are in bold; all predictor variables are included in the model

**Table 3:** Reading bowel screening information: descriptive statistics, adjusted ORs, 95% CIs, *p*-values for variables in the multivariable ordinal logistic regression model

	Amount read of the bowel screening leaflet: n (%)/M (SD)						Adjusted OR, 95% CI	*p*-value
	None of it	Some of it	About half of it	Most of it	All of it	Total	Read more of the leaflet	
Characteristic	(*n* = 13; 4.2%)	(*n* = 44; 14.2%)	(*n* = 14; 4.5%)	(*n* = 63; 20.4%)	(*n* = 175; 56.6%)	(*n* = 309)	(i.e. any amount more)	
Gender								
Male	8 (5.0)	28 (17.5)	7 (4.4)	31 (19.4)	86 (53.8)	160	0.79, 0.50 to 1.27	.338
vs. Female (reference category)	5 (3.4)	16 (10.7)	7 (4.7)	32 (21.5)	89 (59.7)	149		
Ethnicity								
White British	10 (3.5)	41 (14.5)	11 (3.9)	57 (20.2)	163 (57.8)	282	2.58, 1.20 to 5.56	**.015**
vs. Other ethnic groups	3 (11.1)	3 (11.1)	3 (11.1)	6 (22.2)	12 (44.4)	27		
Marital status								
Married or living as a couple	4 (1.9)	23 (11.1)	10 (4.8)	45 (21.7)	125 (60.4)	207	1.65, 0.99 to 2.76	.056
vs. Single, widowed, divorced, or separated	9 (8.8)	21 (20.6)	4 (3.9)	18 (17.6)	50 (49.0)	102		
Highest level of education							Overall: **.003**	
No formal qualifications	8 (7.1)	18 (16.1)	5 (4.5)	27 (24.1)	54 (48.2)	112	0.30, 0.14 to 0.64	**.002**
Approximately Level 1, 2, or 3	4 (3.2)	21 (16.9)	4 (3.2)	27 (21.8)	68 (54.8)	124	0.31, 0.15 to 0.40	**.001**
vs. Approximately Level 4	1 (1.4)	5 (6.8)	5 (6.8)	9 (12.3)	53 (72.6)	73		
Social class grade							Overall: .907	
Grade A or B	3 (3.5)	10 (11.6)	4 (4.7)	13 (15.1)	56 (65.1)	86	0.92, 0.45 to 1.88	.916
Grade C1 or C2	5 (4.1)	11 (9.1)	4 (3.3)	32 (26.4)	69 (57.0)	121	1.06, 0.61 to 1.85	.844
vs. Grade D or E	5 (4.9)	23 (22.5)	6 (5.9)	18 (17.6)	50 (49.0)	102		
Personal diagnosis of cancer								
Yes	2 (5.7)	2 (5.7)	2 (5.7)	10 (28.6)	19 (54.3)	35	1.18, 0.5 to 2.48	.662
vs. No	11 (4.0)	42 (15.3)	12 (4.4)	53 (19.3)	156 (56.9)	274		
Knows someone with cancer								
Yes	8 (3.9)	23 (11.3)	6 (2.9)	37 (18.1)	130 (63.7)	204	1.84, 1.13 to 2.99	**.014**
vs. No	5 (4.8)	21 (20.0)	8 (7.6)	26 (24.8)	45 (42.9)	105		
Bowel screening regularity							Overall: **<.0005**	
Never or very rarely attended…	7 (11.3)	14 (22.6)	4 (6.5)	12 (19.4)	25 (40.3)	62	0.34, 0.19 to 0.59	**<.0005**
Attended some or most of the time…	1 (5.3)	2 (10.5)	2 (10.5)	7 (36.8)	7 (36.8)	19	0.43, 0.18 to 1.04	.061
vs. Always attended before most recent invitation	5 (2.2)	28 (12.3)	8 (3.5)	44 (19.3)	143 (62.7)	228		
Age (in years)	65.5 (2.8)	67.3 (2.6)	65.8 (3.8)	65.8 (2.9)	65.4 (3.3)	65.8 (3.2)	0.92, 0.85 to 0.99	**.029**
Decision-making style								
Avoidant score	13.1 (3.1)	13.5 (3.9)	13.9 (3.8)	13.0 (3.5)	12.6 (3.4)	12.9 (3.5)	0.96, 0.89 to 1.04	.298
Dependent score	15.4 (4.7)	16.5 (3.5)	16.8 (4.6)	16.0 (4.1)	16.5 (3.5)	16.4 (3.7)	1.04, 0.97 to 1.11	.291
Intuitive score	18.1 (3.4)	18.3 (2.8)	18.9 (2.6)	18.8 (2.8)	18.5 (2.6)	18.6 (2.7)	1.10, 1.00 to 1.22	.061
Rational score	18.2 (4.0)	19.3 (2.9)	19.1 (2.1)	19.8 (2.2)	20.0 (2.1)	19.8 (2.4)	1.11, 1.00 to 1.23	**.045**
Spontaneous score	15.5 (4.4)	14.5 (3.6)	14.9 (3.9)	13.5 (3.0)	13.2 (3.4)	13.6 (3.4)	0.93, 0.86 to 1.00	.051

Adjusted ORs and 95% CIs are per unit increase in the case of continuous variables; *p*-values <.05 are in bold; all predictor variables are included in the model

### Predictors of reading the breast screening information leaflet

Analysing predictors of reading all (vs. less than all) of the breast screening leaflet showed that white British participants were more likely to have read all the leaflet than participants from other ethnic groups whereas participants who were older or had never or very rarely attended for screening were less likely to have read it all (compared with participants who had always attended). The five DMS did not seem to be associated with reading all (vs. less than all) of the leaflet (Table 1).

The association with regularity of uptake was consistent in all three sensitivity analyses in which the outcome was dichotomised at different thresholds; ORs ranged from 0.12 to 0.19 for never or very rarely vs. always attended and *p*-values ranged from .001 to .004 (for the comparison of these specific levels) and .003 to .014 (for the variable overall). Associations with ethnicity and age were not reproduced: *p*-values ranged from .187 to .464 for ethnicity (ORs ranged from 1.43 to 1.76) and .205 to.705 for age (ORs ranged from 0.97 to 1.01). No additional predictors appeared to be associated in any of the three models (all *p*-values ≥ .082). Full results are included in Appendix 2.

### Predictors of reading the cervical screening information leaflet

Examining predictors of reading (more of) the cervical screening leaflet showed that participants who very rarely or never attended were less likely to have read more of it than those who always attended. Scores on the measure of rational decision-making were also associated with reading the leaflet: participants with higher scores were more likely to have read more of it. There was moderate evidence against the null hypothesis for social class between grades C1 and C2 vs. grades D and E. However, there was weak evidence against the null hypothesis for the variable overall and so this was not interpreted to be a true association. There was weak evidence against the null hypothesis for the remaining predictor variables (Table 2).

### Predictors of reading the bowel screening information leaflet

As with the previous analyses, regularity of uptake was associated with reading the leaflet: participants who had never or very rarely attended screening in the past were less likely to have read more of it (vs. those who had always attended). This was also the case for older participants and those with a lower level of education (for both those who had no formal qualifications and those who had approximately Level 1, 2, or 3 qualifications vs. those who had approximately Level 4 qualifications). Participants who knew someone with cancer or were White British were more likely to have read more of the leaflet. There was moderate evidence against the null hypothesis for marital status: participants who were married or living as a couple tended to have read more of the leaflet than others. There was weak evidence against the null hypothesis for other demographic predictors and avoidant, dependent, and intuitive DMS. However, participants with higher rational DMS scores were more likely to have read more of the leaflet and there was moderate evidence against the null hypothesis for the spontaneous DMS with the association in the opposite direction (Table 3).

## Discussion

This study, based on a survey of the screening-eligible general public in England, found consistent relationships between past screening participation and engaging with screening information received at the most recent invitation for all three programmes. Specifically, individuals who had very rarely or never been screened in the past were less likely to have read more (or all) of the most recent information leaflet they received, compared with those who had always been screened. This corresponds with the findings of our previous study, which found those who had never undergone bowel cancer screening were much less likely to have read any of the leaflet (for Faecal Occult Blood testing; only 37%) than those who had ever been tested (96% of whom reported having read at least some of the information). The present results extend these findings by demonstrating similar associations for breast and cervical screening, suggesting that there might be related underlying explanations. It is possible that these associations are related to unmeasured characteristics which lead to both declining screening invitations in the first instance and then disregarding information materials at subsequent invitations (i.e. a general disengagement with screening). Relevant factors could therefore include many of the potential determinants of screening uptake such as fatalism [22], low (perceived) risk [23, 24], and greater discomfort thinking about cancer [25]. This might imply that interventions aiming to improve screening uptake, specifically, may also have positive effects in terms of reading screening information at subsequent invitations. However, this approach to improving informed choice alone would have flaws, given that invitees would have already been asked to make at least one prior screening decision, which may not have been informed itself. As a result, it may be preferable to investigate how to increase engagement with screening information at the first invitation. A further related line of research would be to investigate whether increasing engagement also results in invitees making more informed decisions to take part, with potentially positive effects on population health.

We also found (less consistent) relationships for age and ethnicity, in which older and non-white British participants were less likely to read all (or more) of the bowel and breast screening leaflets. Insofar as these reflect genuine associations (given that they were not replicated in the sensitivity analyses for the breast screening leaflet), they may reflect an effect of the number of invitations that people have received (older people will have been invited more often and may have felt less need to re-read the information) and language barriers (non-white British participants may have been less likely to speak English as a first language and so may have found the information less comprehensible). Although screening information leaflets are available in multiple languages, they are less easy to obtain than the English-language default which is sent with all invitations. However, we did not collect data on the language of leaflets that participants received, or participants’ first language, meaning that this may warrant further investigation.

Three possible associations were only observed for the bowel screening leaflet, namely education, knowing someone diagnosed with cancer, and marital status. To some extent, the former result was unexpected, given that no such relationship was observed in our previous study [8]. However, there is evidence that a lack of education is associated with lower health literacy [26] and lower health literacy is associated with less information-seeking and greater effort in reading information about bowel cancer screening [27], so it is plausible that these individuals would be less likely to engage with the leaflet. There is also strong prior evidence that uptake of bowel cancer screening is associated with a family history of the disease (e.g. [12–15]), which often translates to a greater personal risk. The present findings might be a reflection of a generalised concern about cancer due to knowing a relative with a diagnosis, translating to greater engagement with screening information. Finally, although there was only moderate evidence against the null hypothesis with respect to marital status, the observation that participants who were married or living as a couple tended to read more of the leaflet than single, widowed, or divorced participants is concordant with the well-established finding that individuals with a partner are also more likely to be screened [9–11]. It may be possible to increase levels of engagement with information leaflets by posting them not only to invitees but also to their partners, along with encouragement to discuss the topic together.

There was little evidence for most hypothesised associations with DMS scores (e.g. that participants with higher avoidance DMS scores being less likely to read more of the leaflets) but there was some evidence to suggest higher spontaneous DMS scores were associated with reading less of the bowel screening leaflet. One speculative explanation is that this relates to the length of the leaflet, which takes time to read and process, making it less attractive to people who prefer to make quick decisions. Rational scores were associated with reading the bowel and cervical leaflets in the intuitive direction, with lower scores being associated with reading less information. The stated aims of the leaflets are e.g. to give *“information about bowel cancer, and the benefits and risks of bowel cancer screening…to help [people] make an informed choice about taking part”* [6]. This approach may be most acceptable to people who tend to make decisions in this way but it may reduce engagement among those who do not. Consequently, alternative communication strategies may increase engagement with the leaflets among the 23% of participants who reported reading about half of it or less. For example, although leaflets are designed based on a “consider an offer” approach that reduces the extent to which invitees have to weigh up screening attributes [28], the present findings suggest that reducing it further may be beneficial (e.g. with advice on where to access the omitted material using a “gist” approach; [29]). However, this approach may need to be balanced against arguments that a given level of information is ethically necessary [30].

It is not clear why some findings were not apparent across all three programmes. For example, the association between knowing someone with cancer and reading the leaflet could have been hypothesised for the Breast Screening Programme [16, 17]. However, the criteria for eligibility of the three programmes meant that samples were highly dissimilar in terms of age and gender. The three programmes also differ substantially in terms of e.g. how well-established they are and the nature of the target disease, with implications for prior knowledge and risk perceptions.

Limitations relate primarily to the measures used. We were reliant on participants self-reporting the main outcomes and past screening behaviour and these are likely to be vulnerable to social desirability bias and also recall errors, given that they relate to events that occurred up to 5 years ago [31]. This may partially account for differences in the proportion of people reporting reading none of the bowel screening information by Kobayashi et al. (22% [8]) and in this study (4%). There were also differences in reported previous screening participation (69% reported having previously been screened vs. at least 80% in this study). We also did not determine whether participants had absorbed screening information prior to their most recent invitation, either by reading the information leaflet at a previous invitation or by accessing the relevant information another way (e.g. during a consultation with a primary care provider or via the web), and so had chosen not to (re-)read the leaflet. Furthermore, although previously tested and widely used, it is unclear what the real-world implications are for a given DMS score. Finally, the design of the survey and eligibility criteria resulted in smaller samples for the analyses of reading the breast and bowel screening leaflets, which may have resulted in less accurate estimates of associations [32]. This was considered acceptable as part of an exploratory analysis but would require further evaluation using confirmatory designs.

## Conclusions

This exploratory study found consistent positive associations between regular uptake of screening tests in the past and reading screening information leaflets at subsequent invitations for all programmes. We also found positive associations with younger age, white British ethnicity, higher levels of education, knowing someone diagnosed with cancer, and higher rational and lower spontaneous DMS scores for some screening programmes. Implications of these findings include possible merits of reducing barriers to non-English information, and reducing its length and complexity (with information accessible elsewhere instead). These latter two suggestions may also help make information more accessible to invitees who are less inclined to weigh up risks and benefits of screening.Table 4Table 5

**Table 4:** Numbers and percentages of participants excluded from the full sample of 2111 participants, for each analysis of screening information

	n (%)		
Reason for exclusion	Breast	Cervical	Bowel
Inapplicable gender	989 (46.8)	989 (46.8)	N/A
Older or younger than eligible age range	717 34.0)	281 (13.3)	1647 (78.0)
Previous diagnosis of relevant cancer	19 (0.9)	17 (0.8)	2 (0.1)
Non-ordinal highest level of education	33 (1.6)	81 (3.8)	55 (2.6)
No previous screening invitation	35 (1.7)	72 (3.4)	66 (3.1)
Did not see the screening information leaflet at last invitation	16 (0.8)	57 (2.7)	9 (0.4)
Missing data on either dependent or independent variables	27 (1.3)	58 (2.7)	23 (1.1)
Total excluded	1836 (87.0)	1555 (73.7)	1802 (85.4)
Total analysed	275	556	309

**Table 5:** Sensitivity analyses: reading breast screening information (women only): adjusted ORs, 95% CIs, *p*-values for variables in binary logistic regression models

		Amount read of the breast screening leaflet							
		Read at least some of the leaflet		Read at least about half of the leaflet		Read most/all of the leaflet		Read all of the leaflet	
Characteristic		(vs. read none)		(vs. read less than about half)		(vs. read less than most/all)		(vs. read less than all)	
		OR, 95% CI	p	OR, 95% CI	p	OR, 95% CI	p	OR, 95% CI	p
Ethnicity									
	White British	1.66, 0.43 to 6.46	.464	1.43, 0.59 to 3.47	.428	1.76, 0.76 to 4.10	.187	3.28, 1.36 to 7.89	**.008**
	vs. Other ethnic groups								
Marital status									
	Married or living as a couple	1.32, 0.54 to 3.23	.540	0.96, 0.52 to 1.80	.907	0.97, 0.53 to 1.78	.924	0.80, 0.45 to 1.42	.450
	vs. Single, widowed, divorced, or separated								
Highest level of education		Overall: .452		Overall: .913		Overall: .791		Overall: .150	
	No formal qualifications	0.44, 0.10 to 1.90	.269	1.09, 0.44 to 2.68	.856	0.85, 0.36 to 2.01	.704	0.44, 0.19 to 1.01	.052
	Approximately Level 1, 2, or 3	0.44, 0.12 to 1.64	.222	1.18, 0.54 to 2.60	.683	1.07, 0.50 to 2.29	.861	0.60, 0.29 to 1.24	.168
	vs. Approximately Level 4								
Social class grade		Overall: .689		Overall: .925		Overall: .782		Overall: .876	
	Grade A or B	0.73, 0.21 to 2.59	.631	1.18, 0.47 to 2.98	.731	0.77, 0.31 to 1.89	.565	0.90, 0.40 to 2.07	.811
	Grade C1 or C2	1.23, 0.43 to 3.54	.706	1.01, 0.50 to 2.06	.977	0.79, 0.39 to 1.59	.508	1.08, 0.57 to 2.05	.811
	vs. Grade D or E								
Personal diagnosis of cancer									
	Yes	0.70, 0.17 to 2.84	.612	1.25, 0.39 to 4.04	.708	1.11, 0.37 to 3.32	.846	0.90, 0.35 to 2.35	.834
	vs. No								
Knows someone with cancer									
	Yes	0.66, 0.24 to 1.86	.435	0.71, 0.36 to 1.40	.328	0.90, 0.48 to 1.69	.750	1.23, 0.69 to 2.20	.493
	vs. No								
Breast screening regularity		Overall: **.008**		Overall: **.014**		Overall: **.003**		Overall: **.014**	
	Never or very rarely attended…	0.12, 0.03 to 0.48	**.003**	0.19, 0.06 to 0.58	**.004**	0.14, 0.44 to 0.47	**.001**	0.16, 0.04 to 0.63	**.009**
	Attended some or most of the time…	1.30, 0.15 to 11.04	.812	0.65, 0.21 to 2.03	.458	0.43, 0.14 to 1.26	.122	0.43, 0.14 to 1.30	.135
	vs. Always attended before most recent invitation								
Age (in years)		1.01, 0.95 to 1.09	.705	0.97, 0.92 to 1.02	.205	0.98, 0.93 to 1.03	.352	0.96, 0.92 to 1.00	**.048**
Decision-making styles									
	Avoidant score	1.13, 0.99 to 1.30	.082	0.97, 0.89 to 1.06	.523	0.94, 0.87 to 1.03	.176	0.97, 0.89 to 1.05	.452
	Dependent score	1.02, 0.91 to 1.14	.761	1.00, 0.92 to 1.09	.973	1.01, 0.93 to 1.09	.876	1.03, 0.96 to 1.11	.434
	Intuitive score	0.97, 0.82 to 1.13	.671	1.08, 0.96 to 1.21	.191	1.09, 0.98 to 1.22	.116	1.01, 0.91 to 1.12	.892
	Rational score	1.02, 0.86 to 1.20	.853	0.99, 0.88 to 1.11	.801	0.98, 0.88 to 1.09	.722	1.08, 0.97 to 1.19	.151
	Spontaneous score	0.92, 0.81 to 1.04	.169	0.99, 0.91 to 1.08	.861	0.98, 0.90 to 0.90	.653	1.03, 0.95 to 1.11	.528

ORs and 95% CIs are per unit increase in the case of continuous variables; *p*-values <.05 are in bold

## Abbreviations

ABACUS: Attitudes, Behaviour and Cancer UK Survey
BTEC: Business and Technology Education Council
CI: Confidence interval
DMS: Decision-making style
GCSE: General Certificate of Secondary Education
NHS: National Health Service
ONC: Ordinary National Certificate
OR: Odds ratio
PAPM: Precaution Adoption Process Model
SD: Standard deviation
SPSS: Statistical Package for the Social Sciences
TNS: Taylor Nelson Sofres
UK: United Kingdom
